# Detection of anomalous spatio-temporal patterns of app traffic in response to catastrophic events

**DOI:** 10.1140/epjds/s13688-025-00546-w

**Published:** 2025-05-06

**Authors:** Sofia Medina, Shazia’Ayn Babul, Timothy LaRock, Rohit Sahasrabuddhe, Renaud Lambiotte, Nicola Pedreschi

**Affiliations:** Mathematical Institute, University of Oxford, Oxford, UK

**Keywords:** Data visualization, Mobile applications, Event detection, Disaster response, Urban mobility, Computational social science, Spatiotemporal phenomena, Patterns in data

## Abstract

In this work, we uncover patterns of usage mobile phone applications and information spread in response to perturbations caused by unprecedented events. We focus on categorizing patterns of response in both space and time, tracking their relaxation over time. To this end, we use the NetMob2023 Data Challenge dataset, which provides mobile phone applications traffic volume data for several cities in France at a spatial resolution of 100 $m^{2}$ and a time resolution of 15 minutes for a time period ranging from March to May 2019. We analyze the spread of information before, during, and after the catastrophic Notre-Dame fire on April 15th and a bombing that took place in the city centre of Lyon on May 24th using volume of data uploaded and downloaded to different mobile applications as a proxy of information transfer dynamics. We identify different clusters of information transfer dynamics in response to the Notre-Dame fire within the city of Paris as well as in other major French cities. We find a clear pattern of significantly above-baseline usage of the application Twitter (currently known as X) in Paris that radially spreads from the area surrounding the Notre-Dame cathedral to the rest of the city. We detect a similar pattern in the city of Lyon in response to the bombing. Further, we present a null model of radial information spread and develop methods of tracking radial patterns over time. Overall, we illustrate novel analytical methods we devise, showing how they enable a new perspective on mobile phone user response to unplanned catastrophic events and giving insight into how information spreads during a catastrophe in both time and space.

**Supplementary Information:**

The online version contains supplementary material available at 10.1140/epjds/s13688-025-00546-w.

## Introduction

Understanding how information propagates during and after catastrophic events is an active field of investigation [1–5]. The granularity of mobile phone traffic volume data, at both the temporal and spatial scales, provides insight into the intricacies of human behavior. Mobile phone datasets thus enable large-scale data driven analysis applied to a wide range of areas including social network analysis [6], population dynamics [7], urban structure [8], public health [9], and disaster response [10]. Social media and online resources have been used to categorize and track the duration and intensity of responses to events and breaking news stories [3–5, 11, 12]. Research has also illustrated behavioral patterns that can both characterize and distinguish between planned and unplanned events [13, 14]. While most studies on social media or app usage have focused on a single type of online activity [3, 13–15], recent work has emphasized the importance of cross-platform analysis to account for platform-specific effects [16, 17]. Further, prior studies of information spread generally focus on the relationships between users in social-network space [18–20]. However, some works investigate the geo-spatial dependence of spread of information over social media [21, 22].

In this work, we analyze mobile phone traffic volume data to understand how the temporal and spatial usage of different applications are perturbed in the aftermath of an unprecedented event and how these usage patterns evolve over time. We focus on how the aggregated usage patterns of a variety of mobile phone applications respond to the same single event. To this end, we use the NetMob2023 Data Challenge dataset [23] which provides mobile phone usage data for several cities in France for a range of applications over a period of time ranging from March to May of 2019 at a spatial resolution of 100 $m^{2}$ and a time resolution of 15 minutes. We investigate dynamics and patterns arising from catastrophic events, most notably, one of the most extreme events occurring during this period: the fire at the Notre-Dame cathedral in Paris and the collapse of its historic spire.

We analyze the spread of information before, during, and after the Notre-Dame fire using the volume of data uploaded and downloaded to different mobile applications as a proxy for information transfer. The methods we develop take advantage of this data to show how information spreads during a major unplanned event and can be extended to characterize mobile phone user response to other categories of events, both planned and unplanned. We demonstrate this by extending our spatial analysis to find consistent results during a second catastrophic event, a bombing in Lyon, France.

We present two kinds of analysis. First, we spatially aggregate data within each city and study the time-series of app traffic per city for a select subset of the available apps. We then use a simple anomaly detection method to determine whether traffic volume for each app spiked after the Notre-Dame fire, when those spikes occurred, and for how long. Next, we use information about these spikes in traffic volume to cluster apps based on how they were used during the event to uncover patterns in how application use changed both within the area local to the event and across other geographic regions. Our results show that applications have overall similar use patterns across cities. However, we highlight variations in app traffic behavior across different cities that suggest the existence of city-specific user preferences. Additionally, we find that a fine-grained description of application function is a more accurate predictor of clustering behavior.

Second, we analyze how traffic spikes were distributed spatially throughout the city of Paris over time as news of the fire spread and mobile phone users sought more information through social media, apps, and streaming services. We find that the abnormally elevated traffic appears to spreads radially from the center of the catastrophe. In order to quantify *how radial* is the spreading of above baseline app traffic in response to catastrophe, we construct an optimally radial null model. The comparison of the original data with the null model allows us to capture the deviation (or accordance) of empirical data from perfectly radial spreading patterns. We thus show that the spreading of abnormal app traffic, for the app Twitter, is indeed radial following the start of the fire. This is consistent with other literature [22], as well as our own extended analysis to a bombing occurring in Lyon, France. We further propose methods for quantifying this radial pattern of the information spread, noting that it this pattern of information spread also extends to planned events.

This paper is organized as follows. In the next section, we describe the NetMob dataset that we work with in this study, as well as our methodology for both temporal and spatial traffic volume pattern analysis. Then, in Sect. 3, we present the results of applying our proposed methodology to the dataset. Finally, in Sect. 4, we discuss in detail the implications and conclusions of our results and some directions for future research.

## Methods

In this section, we describe the methodology pertaining to the detection and analysis of patterns of spikes in application traffic volume both temporally and spatially.

### Background & data

The Notre-Dame cathedral, built during the 12th and 13th centuries, and has long been recognized as an emblem of French society. Located in the center of Paris, it is a highly popular tourist attraction, as well as a religious site. On April 15th, 2019, the roof of the Notre-Dame cathedral caught on fire and was severely burned. The fire broke out in the attic of the cathedral at 18:18 [24]. The occupants of the building, including tourists and worshipers taking mass, evacuated minutes later, at 18:20 [25], when a fire alarm sounded. Smoke was first visible by 18:43 from Paris’ Left Bank [25], with firefighters officially called to the cathedral at 18:51 [26] when cathedral workers discovered the fire. The historic spire of the cathedral collapsed at 19:50 [24]. Given that the fire occurred at one of the most historic buildings in Europe, news spread quickly through both social media and traditional news media sources.

The dataset we will analyze is the NetMob2023 Data Challenge dataset, which provides the traffic volume for 68 mobile applications in 20 cities in France at a spatial resolution of $100~m^{2}$ and a time resolution of 15 minutes for 77 days, ranging from March 16th to May 31st 2019 [23]. The data contains both uplink (transmissions to the cell tower) and downlink (transmissions from the cell tower) traffic. Since both uplink and downlink represent usage of an application and different applications will have different volumes of each depending on their function and implementation, we analyze both directions simultaneously by summing the two.

We limit our analysis to data from the largest six largest cities in the dataset, i.e., Paris, Marseille, Lyon, Montpellier, Rennes, and Strasbourg. We note that the dataset does not contain information regarding content transferred along the cellular network.

In our analysis, we consider a subset of applications in the dataset after sorting for relevance (e.g. exclusion of mobile phone games) and ability to distinguish app traffic as being in some form related to the fire (e.g. exclusion of general internet browsing such as Yahoo). A full list of applications considered, as well as excluded, is given in the Supplementary Materials.

### Detecting spikes in app traffic volume

In our dataset, each city *c* is represented spatially by a tiling (or tessellation); we refer to the tiles of a city *c* as a list $w_{c}=1,\dots ,n_{c}$, where $n_{c}$ is an integer corresponding to the total number of tiles in that city. We then denote the volume of traffic for app *α* in city *c* at tile $i\in w_{c}$ at time *t* on day *d* as $\rho _{\alpha , c, i}(t, d)$.

We form a temporal description of the traffic volume by spatially aggregating the traffic for each app over the entire city for each day:

1 $$ \rho _{\alpha , c}(t, d) = \sum _{i \in w_{c}} \rho _{\alpha , c, i}(t, d), $$

recovering a time series representing the total traffic in the city at each 15 minutes time interval *t* for a day in the dataset.

We utilize these time series to calculate baseline statistics against which to measure abnormal app traffic. Since the fire of the Notre-Dame cathedral occurred on Monday April 15th, we construct the baseline and standard deviation based on app traffic from the weekdays, Monday through Thursday, preceding the fire in the dataset: March 18th to April 11th 2019. We exclude Fridays and weekends as we expect they will have more heterogeneous application traffic patterns. We also confirmed that no national holidays coincided with our baseline period. We denote the mean traffic volume over this period as

2 $$ \mu (\alpha ,c,t) = \langle \rho _{\alpha , c}(t, d) \rangle _{d} . $$

The value of $\mu (\alpha ,c,t)$ at each time *t* corresponds to the average traffic volume for the app *α* during the 15 minute window *t*. We also compute the standard deviation for each tile and time and denote it $\sigma (\alpha , c, t)$. For convenience, from here on we will drop the indices for app and city, and simply use $\mu (t)$ and $\sigma (t)$ to represent the average and standard deviation for any combination of app and city over the baseline period. Similarly, we will use $\rho (t,d)$ to refer to a time series on a specific day.

We now define the *distance from baseline* at time *t* on day *d* as the following modified z-score,

3 $$ D(t,d) = \frac{\rho (t,d) - (\mu (t) + 2\sigma (t))}{\mu (t) + 2\sigma (t)}, $$

indicating the value of traffic volume in relation to two standard deviations from the baseline mean of traffic.

Finally, we define a spike in the traffic of an app as a period of at least 3 consecutive time points $[s_{start},s_{end}]$ where $D(t)>0$, in order to account for noise. For symmetry, we define the end of a spike as a period of three consecutive time points $[s_{end}+1,s_{end}+3]$ where $D(t)<0$.

### Temporal clustering

We expect that spikes in application traffic volume will exhibit different characteristics in terms of duration, amplitude and position in time. In this section we define a set of features on the distance from the baseline time series $D(t)$ of an application and show how we use these features as inputs to a clustering algorithm to characterize apps with similar traffic spiking patterns.

We break our analysis into two days, first clustering immediate reactions to the fire as application behaviors on the day of the fire and then looking at longer term behaviors of applications the day after the fire. We introduce an overnight gap on the day after the fire from midnight to $7:00$, since baseline weeknights have small variation compared to weekdays, meaning smaller standard deviations and more noise using our anomaly detection method. Considering that the smoke during the fire of Notre-Dame is first visible at 18:43, we denote a spike occurring from $18:45$ of April 15th to the end of that day as spike 1, and a spike occurring from $7:00$ to $24:00$ of April 16th as spike 2. We compute at most one spike per day, with only a first possible spike being considered.

We therefore end up with at most two spikes, one for the day of the fire and one for the day after, for each city and app combination. We then compute the following 5 features about each spike:

- ($s_{i}^{start}$, $s_{i}^{end}$): the starting and ending times of the *i*th spike
- $s_{i}^{duration}$: the duration of the spike
- $s_{i}^{max}$: the maximum value of $D(t)$ during the spike
- $s_{i}^{aggregate}$: The sum of all $D(t)$ values during the spike

Using these features we represent the traffic time series of each app on the day of and day after the fire as two features vectors of length 5, and we normalize the features vectors by the maximum and minimum values for spiking apps within each feature for the day.

Using the feature vectors computed above representing changes in app traffic volume, we perform a K-means clustering of the apps based on their feature vector representations. We find empirically that, due to our time period of interest, the number of apps that spike in Paris is much larger than in the other cities in the dataset. Since we would like to understand if changes in application traffic are different in the city of incidence of catastrophe, we decided to cluster apps in Paris separately from all other cities. Thus, we compute clustering for the following groupings independently: Paris on April 15th, Paris on April 16th, other cities on April 15th, and other cities on April 16th. We choose number of clusters $k=5$ by computing the Sum of Squared Errors over values of *k* and manually inspecting the curves. For a consideration of traffic spikes as a single event across both days as a 10 dimension features vector, see Supplementary Materials.

### Spatial analysis

In this section we describe our methodology for understanding spatial patterns in changing app traffic volume in response to major events. We first describe how we translate the concept of spiking traffic introduced above into the spatial dimension, then describe a null model against which we can compare empirical spatial traffic patterns.

Our spatial analysis is restricted to the traffic response to the fire of Notre Dame for the application Twitter in the city of Paris. Paris is considered as this is the city of incidence of the fire, allowing for measurement with respect to the epicenter of catastrophe. Only Twitter is considered as this application experiences a both strong and long term response, giving the possibility of analyzing its behavior over time.

#### Traffic spikes in space

We first recall that the Netmob2023 Data Challenge dataset has a spatial resolution of 100 $m^{2}$ given as a tiling of the city of interest. Hence, all spatial analysis can be conducted at this spatial resolution. Here we will consider only the tile-wise time series of $\rho _{\alpha , c, i}(t, d)$ (see Equation (1)) for the city of Paris (*c*= Paris), and for Twitter (*α*= Twitter). Now for *each tile* $i\in w_{Paris}$ we define the tile-wise Twitter time-series as $\rho _{i}(t, d)$.

We use these tile-wise time series to calculate a baseline mean and standard deviation for the traffic for each spatial tile. Following the same methodology as described above for the city-wide time series, we construct our baseline statistics based on Twitter traffic on the weekdays Monday through Thursday from March 18th to April 11th, again excluding Fridays and weekends as we expect they will have different patterns. We denote the mean traffic volume over this period for a tile *i* similarly as:

4 $$ \mu _{i}(t) = \langle \rho _{i}(t, d) \rangle _{d} . $$

The value of $\mu _{i}(t)$ at each time *t* now corresponds to the average traffic volume for Twitter during the 15 minute window *t* in tile *i* of the spatial grid of the city of Paris. We also compute the standard deviation for each tile and time and denote it $\sigma _{i}(t)$.

We can now define the tile-wise *distance from baseline* at time *t* on day *d*, in tile *i* as the following modified z-score:

5 $$ D_{i}(t,d) = \frac{\rho _{i}(t,d) - (\mu _{i}(t) + 2\sigma _{i}(t))}{\mu (t) + 2\sigma (t)}. $$

We compute $D_{i}(t)$ for Twitter on each tile *i* of the city of Paris starting from $t=\textrm{18:45}$ on April 15th, the day of the fire, until $t=\textrm{24:00}$.

#### Quantifying spatial effects

We are interested in how information propagates across applications with respect to the source of the catastrophic event. That is: what spatial patterns emerge in response to catastrophic events, how can we quantify these patterns, and how do they evolve temporally? Since the concept of physical distance does not exist in the virtual application space, it is of particular interest to understand how quickly information propagates across the internet and how this translates to analogous physical spatial patterns.

Our analysis of physical spatial patterns takes inspiration from work by Bagrow et. al that considers the relationship between anomalous traffic volume and the distance from the epicenter of the catastrophe [22]. We begin by constructing concentric square shells of increasing radial size centered on the epicenter, with radius being the diagonal of each square and the distance from the epicenter measured in kilometers. A shell is defined as the collection of all of the tiles included in the square of the current radius and excluding all of the tiles from the previous radial distance, in essence a square annulus. For each shell, we sum the total anomalous traffic, $\sum _{i \in r} D_{i}(t)$, within the shell and normalize by the number of tiles within the specified shell, following [22]. This gives the following measure that shows the mean tile-wise volume of application traffic as a function of the radius:

6 $$ \Delta D(r) = \cfrac{\sum _{i \in r_{m}} D_{i}(t)}{\sum _{r_{m}} i} \textrm{ for } m \in 0,1,2,\dots ,M . $$

The radius zero is defined in [22] as the single tile at the epicenter of the event. In our analysis, we consider a maximum shell radius to be the value of the last tile before exiting the defined city tiling in any direction. This consideration is sufficient in our analysis to recognize spatial patterns, however, this directional consideration may be adjusted, particularly for events which may occur on the edge of a defined city tiling where radial shells will quickly exit the city in a certain direction.

We now have a measure of abnormal spatial traffic at each radius, which allows us to quantify changes in traffic at all radial distances. We posit that given Equation (6), the radial outward spread from the epicenter would correspond to a volume of the above-baseline app traffic that decays as a function of the distance from the epicenter.

We are interested in how the radial spread of information relaxes in the immediate aftermath of a major event. Thus, we consider the ‘instantaneous’ function of Eq. (6) at each measured time point. This fine-grained approach to understanding radial spread allows us to analyze how radial patterns evolve and relax over time after a major event.

We now have a measure of radial spread that can be analyzed at each time point. We can use this measure to ask how radial is the spread of information and how quickly do abnormal application traffic patterns relax following a major event. We use linear regression to recover a best-fit slope for the data at each time-point, tracking how the slopes of the recovered fits change throughout the course of the event. The radial decay is stronger as the value of the slope decreases.

We further explore the comparison of the data spread and its changes to a radial spread using Kullback-Leibler (KL) divergence. We first measure the KL divergence of each instantaneous snapshot in the form of Eq. (6) to the aggregated function of Eq. (6) two hours after the event, showing how the data changes from before, during, and after the fire as compared to its two hour change immediately following the catastrophe.

We then extend our analysis by constructing a null model of optimal possible radial spread given the data. The null model is constructed for a time point by considering all values of abnormal traffic for spatial tiles in the maximum radius searched in that time point. These values are then sorted by maximum value outward from the epicenter. That is, the maximum value of all tiles in the snapshot is assigned to the epicenter of the event, the next *n* highest values are then assigned to the *n* tiles within the first radius considered away from the epicenter, and so forth. We can then calculate a function of the form of Eq. (6) on this optimal radial spread according to the data, which we compare to the true data distribution at each point in time by measuring the KL divergence between the two distributions. Finally, we plot the measured KL divergences over time between the instantaneous snapshots to both the aggregate and null models, tracking the extent to which the spread of information approximates a radial spreading pattern that relaxes from the radial state over time.

## Results

We now describe the results of applying our proposed methodology to the NetMob2023 Data Challenge dataset. The broad aim of this analysis is to capture how the volume of traffic of different applications varies in response to a catastrophic event and whether changes in traffic volume within the city of Paris are different from those found in other cities during the fire of Notre-Dame. We investigate the patterns of application traffic response that emerge, first temporally and then both temporally and spatially.

### Anomalous application traffic and behavioral clusters

We identify spiking behavior of applications through their time series of *distance from baseline* $D(t,d)$, at time *t* on day *d* (see Methods for formal definition). We thus consider a spike in the traffic of an app a time-span of at least 3 consecutive time points where $D(t,d)>0$.

In Table 1, we list the 11 apps that present spikes of traffic in response to the fire in the city of Paris; we also identify subsets of the same apps whose traffic volume spikes above baseline in the cities of Lyon, Marseille, Montpellier, Rennes and Strasbourg. Abnormal app traffic volume spikes, both in Paris and in other French cities, are consistent with the hypothesis that changes in mobile internet traffic volume correspond to real-time reactions to catastrophic events. Interestingly, the sets of applications that spike in cities other than Paris are always a subset of the applications that spike in the French capital, where the fire occurred.

**Table 1:** Comprehensive table of all apps that spike in Paris (Pa), Marseille (Mrs), Lyon (Ly), Montpellier (Mtp), Rennes (Rn) and Strasbourg (Sg)

Application	Pa	Mrs	Ly	Mtp	Rn	Sg
Apple Video	✓		✓	✓		✓
Apple iCloud	✓					
DailyMotion	✓			✓		✓
Facebook	✓			✓		✓
Facebook Live	✓					
Facebook Messenger	✓	✓	✓	✓	✓	✓
Instagram	✓		✓	✓		
Molotov	✓		✓	✓		✓
Periscope	✓	✓	✓	✓	✓	✓
Twitter	✓	✓	✓	✓	✓	✓
WhatsApp	✓			✓		

We aim to identify groups, or clusters, of similar application traffic spiking patterns that correspond to various responses to the event or shed light on the ways in which the news of the fire is spread across platforms. Our analysis includes both the hours immediately following the fire of Notre-Dame on April 15th as well as patterns of traffic on the following day.

#### Patterns on the day of fire

First, we compute the five features that describe the spiking patterns observed in the individual traffic volume time series of each application (see definitions in Methods): $s_{1}^{start}$, $s_{1}^{end}$, $s_{1}^{max}$, $s_{1}^{duration}$ and $[s_{1}^{start},s_{1}^{end}]$.

We then perform a K-means clustering on the spiking-features vectors of all apps that spike within the city of Paris (Fig. 2.a), and all the apps that spike in the other selected French cities (Fig. 2.b). For a full description of the values of the selected features for each application, see Supplementary Materials (Supplementary Figures 12, 14).

In Fig. 2 each cluster consists of applications whose traffic time series display similar responses to the event as characterized by their feature vectors. We represent the typical response pattern of apps assigned to each cluster as a normalised radar plot of the *centroid* of each cluster (Fig. 2.a, left and Fig. 2.b, left). The radial axes of the radar plot correspond to the previously defined spiking features. The suffix 1 in $s_{1}$ refers to *first* day, or day of fire. We note that the spiking features are normalized with respect to its minimum and maximum values, across all apps, found for the city of Paris (Fig. 2.a), and for the other cities (Fig. 2.b). Next to each radar plot, we show a representative time series of $D(t,d)$ of one of the applications assigned to the relevant cluster (Fig. 2.a, right and Fig. 2.b, right).

In the city of Paris, we recover from our clustering methods three clusters of applications with similar behavioral patterns and two outliers containing the singular behavior of an application. The applications in each cluster are given in Table 2. Cluster 1 is characterized by low-amplitude, long spikes with late start times relative to other clusters (high $s_{1}^{start}$), yet sustained until the end of the day (high $s_{1}^{duration}$ and $s_{1}^{end}$). Apps in this cluster are thus characterized by a delayed and moderate, yet sustained, response to the spreading of the news of the fire. Cluster 2 is composed of apps with a similarly delayed spike of relatively moderate amplitude, but with short, rather than sustained, duration. The response to the fire is closer to the collapse of the spire, rather than the moment in which the fire becomes visible, yet the perturbation of the traffic time series of apps in this cluster is the shortest with respect to the other clusters. Applications in Cluster 3 are characterized by long, low-amplitude spikes, with very early start times (low $s_{1}^{start}$). Apps in this clusters, such as Instagram, see a rise above baseline of their traffic volume as soon as the fire becomes visible from the outside of the cathedral; the traffic volume stays persistently above baseline throughout the rest of the day, yet the intensity of such anomalous activity is moderate.

**Table 2:** Comprehensive table of all apps per cluster, in Paris on the day of fire, 15th April 2019. See Fig. 2

Cluster 1	Cluster 2	Cluster 3	Cluster 4	Cluster 5
DailyMotion	Apple Video	Apple iCloud	Periscope	Twitter
Facebook Live	Molotov	Facebook		
	WhatsApp	Facebook Messenger		
		Instagram		

The first outlier cluster, Cluster 4 includes one app with a distinctive behavior, Periscope, which is a live video streaming app. The traffic time series for Periscope has a massive spike (10 times larger than average over the previous weeks in the same time points) at 19:00, *i.e.*, in the 15 minutes immediately following the smoke. The amplitude of the spike then decreases rapidly after 45 minutes, yet it stays above the baseline activity until the end of the day. This pattern of usage regarding Periscope is unique to the application, suggesting a strong preference among users in the dataset for using Periscope to stream or watch video of the event.

Finally, the second outlier cluster, Cluster 5, contains only the discussion-based social media platform Twitter. Twitter traffic in Paris is characterized by a well sustained high-amplitude spike that suggests the importance of the platform to response to the Notre-Dame catastrophe.

In other cities, we also recover 5 clusters of applications, giving further insight into user preference within and across cities. The applications and their respective clusters are illustrated in Table 3. We note that although the same application in different cities mostly belong to the same clusters, there are some differences. We also obtain one outlier cluster that contains a single application, Instagram in Lyon, which demonstrates completely distinct behavior from both other application usage in Lyon and Instagram usage in other cities. This emphasizes the conclusion that variations in application traffic behavior are related to both city-specific user preferences as well as more general usage dynamics.

**Table 3:** Comprehensive table of all apps per cluster spiking on the day of fire, 15th April 2019, for all other cities: Marseille (Mrs), Lyon (Ly), Montpellier (Mtp), Rennes (Rn) and Strasbourg (Sg). See Fig. 2

Cluster 1	Cluster 2	Cluster 3	Cluster 4	Cluster 5
Instagram Ly	Facebook Messenger Mrs	Apple Video Sg	Periscope Mrs	DailyMotion Mtp
	Facebook Messenger Sg	Apple Video Mtp	Periscope Sg	Facebook Mtp
	Facebook Messenger Ly	DailyMotion Sg	Periscope Ly	Instagram Mtp
	Facebook Messenger Mtp	Facebook Sg	Periscope Mtp	Twitter Mrs
	Facebook Messenger Rn	Molotov Sg		Twitter Sg
		Molotov Ly		Twitter Ly
		Molotov Mtp		Twitter Mtp
		Periscope Rn		
		Twitter Rn		
		WhatsApp Mtp		

Cluster 2 comprises apps characterized by early and sustained spikes, that relax back to baseline well before the end of the day. Cluster 3 groups together apps whose traffic is perturbed early in response to the fire, however the amplitude and duration of such spikes are very low in comparison with apps in the other clusters. Cluster 4, which includes the application Periscope for almost all other cities, experiences a short but intense spike of large amplitude, coherently with what is observed in Paris. Finally, Cluster 5 is composed of applications experiencing elevated levels of application traffic that last until the end of the day.

While these patterns have slight variations between the 5 types of behavior in Paris and the 5 types of behavior in other cities, there are notable similarities in user usage for applications in response to catastrophe. This is particularly relevant to the outlier clusters in the city of Paris and how they translate to the across-city clusters. Both in Paris and in other cities, the live video streaming app Periscope is characterized by a very large, yet brief, spike in usage, taking place shortly after the start of the fire. Like in Paris, a single cluster (Cluster 4 of other cities) is characterized only by application traffic patterns of the app Periscope across 4 other cities. This result shows the consistency of user interaction with this specific platform, showing that user tendencies can conform across for an application with a narrow scope, such as live video streaming. Patterns that are similar the French capital and in the other cities, are also evident in the behavior of Twitter. For 4 of 5 cities, Twitter clusters into Cluster 5. However the participation of the Twitter application in many cities in this cluster is shared with other apps, unlike in the case of Paris where Twitter is an outlier that does not cluster with other applications. This showcases the existence of general trends in app usage across cities as well as city-specific user preference effects. Applications with the behavior pattern of the cluster primarily represented by Twitter undergo a large spike after the start of the fire, have a sharp increase in traffic until the collapse of the spire of the cathedral, and remain persistently well-above baseline during the rest of the day. This is true for both Paris and other cities. For both Paris and other cities there is also a cluster (Cluster 1 in both Figs. 2.a and 2.b) with shallow spikes that start relatively late with respect to other applications.

We further consider whether applications in the other cities, excluding Paris, cluster similarly across these cities. We find that while this is mostly the case, this phenomenon is not exclusive. Applications that spike in multiple cities tend to cluster together, for example, Facebook Messenger clusters together for all the cities. However, there is some slight variation of application usage based on within-city user behavior. This is evident in that there are apps that spike in some cities, and not others. For example, Instagram and Molotov spike in some cities, but not in all. Furthermore, there is some slight variation in how the apps cluster, such as how Periscope primarily clusters together, with the exception of the city of Rennes, which is assigned to Cluster 3. The same can be seen with Daily Motion, which appears for only 2 of the other 3 cities considered, in separate clusters. These results suggest the strong affect of city specific user-preference.

We find that applications with the same *‘function’*, such a social media platforms, can exhibit very different response patterns. One might have better success in predicting app response patterns by a more fine grained type such as, messaging, broadcasting, image sharing, live video, etc. For example, the reaction-based platforms Facebook Live and DailyMotion cluster together, but don’t cluster with other platforms that could generally be considered social media.

#### Patterns on the day after fire

We now consider the long-term behavior of applications and their relaxation from abnormal traffic both in Paris and in other cities by considering app traffic on the day after the fire. We begin our analysis at 7:00 on the following day to account for small baseline standard deviations during the night. In Fig. 3 we show app clustering for Paris (Fig. 3.a) and other cities (Fig. 3.b) on the day after the fire of Notre-Dame. The applications and their respective clusters are illustrated in Table 4 and Table 5. We note that for most applications, there is a quick general relaxation of spiking behavior, that is, the abnormal app traffic does not last into the day after the fire. However, there are few exceptions, particularly in the city of Paris. Twitter, which appears in Cluster 3, had elevated traffic levels throughout the second day, possibly due to new user content (discussion) being generated on the forum, unlike an image based platform like Instagram. Notably, there is some spiking behavior in clusters both for Paris and for other cities in the evening, as can be seen in Clusters 3, 4, and to a lesser extent, Cluster 1. This is particularly notable in the spiking of Periscope. We posit that this spiking, such as that of the video application Periscope and the messaging platform Whatsapp are associated to the organizing of and sharing of content concerning a vigil held in response to the fire at the Cathedral of Notre-Dame, which occurred approximately 24 hours after the fire and lasted into the night [27].

**Table 4:** Comprehensive table of all apps per cluster, in Paris on the day after the fire, 16th April 2019. See Fig. 3

Cluster 1	Cluster 2	Cluster 3	Cluster 4	Cluster 5
Apple iCloud	Molotov	Apple Video	DailyMotion	Facebook Live
Facebook	Periscope	Twitter	WhatsApp	Facebook Messenger
Instagram

**Table 5:** Comprehensive table of all apps per cluster, on the day after the fire, 16th April 2019, in the other cities: Marseille (Mrs), Lyon (Ly), Montpellier (Mtp), Rennes (Rn) and Strasbourg (Sg). See Fig. 3

Cluster 1	Cluster 2	Cluster 3	Cluster 4	Cluster 5
Periscope Sg	DailyMotion Sg	Molotov Ly	Apple Video Ly	Apple Video Sg
Periscope Rn	DailyMotion Mtp	Molotov Mtp	Apple Video Mtp	Facebook Sg
	Facebook Mtp	Twitter Mtp	Facebook Messenger Mrs	Facebook Messenger Sg
	Periscope Mrs		Periscope Ly	Facebook Messenger Ly
	Periscope Mtp			Facebook Messenger Mtp
				Facebook Messenger Rn
				Instagram Ly
				Instagram Mtp
				Molotov Sg
				Twitter Mrs
				Twitter Sg
				Twitter Ly
				Twitter Rn
				WhatsApp Mtp

Additionally, we note that there no longer exist single-application clusters in Paris, as distinct behaviors collapse into more general long-term patterns. We found that for other cities, with the exception of Periscope in two cities, spiking patterns on the day after the fire were low and few and thus difficult to parse from general random spiking unrelated to the fire. Cluster 5 in both Paris and in other cities, are the clusters containing all apps that do not exhibit any spike on the day after the fire.

### Spatial response

In this section we investigate how changes in traffic volume propagate with respect to the geographic location of the source of the catastrophic event. We consider the association between information spread, using traffic volume as a proxy measure, in both time and intensity, and the distance from the epicenter of the catastrophe. We identify and quantify spatial patterns of above baseline traffic volume and their evolution over time. We define spatial above-baseline traffic, $D_{i}(t)$, tile-wise as the difference in application traffic volume in that tile from two standard deviations above its weekday mean (see Methods for formal definition). For this analysis, we only take into account the application Twitter in the city of Paris, as it undergoes a sustained, abnormally elevated app traffic volume in the city of incidence.

In Fig. 4 we plot abnormally elevated application traffic volume, $D_{i}(t)$, in the metropolitan area of Paris on the day of the fire from 18:00 to several time points following the fire. We note how, before the fire, the above-baseline traffic is spatially distributed as random noise. However, after the fire starts, we detect an approximately *radial*, outward spreading pattern emanating outward from the Notre-Dame Cathedral. This pattern appears to persist until the end of the day, relaxing over time in both intensity and traveled radial distance.

Given that mobile phones users at any physical distance could in principle access information at the same time, a pattern of radial spread that appears dependent on physical distance is not guaranteed to emerge. We thus quantitatively address the pattern of outward radial spread, with the cathedral as its origin. We track how the intensity of anomalous traffic, aggregated over the course of 2 hours after the start of the catastrophe, changes with the distance from the epicenter, *i.e.*, the cathedral, building on the work of [22]. We use the change in traffic volume in subsequent squares of increasing diagonal length normalized by the number of tiles within the considered distance, $\Delta D(r)$, to describe this relationship. That is, we analyze how aggregated traffic, $D(r)$, within some defined area changes as the radial distance from the epicenter increases (see Methods for further details). Noting that a radial spread of intensity of abnormal traffic outward from the epicenter would be approximated by a decaying function of this form (see Methods, Eq. (6)), we find that in general the spread of traffic volume after the fire seems to follow a dependence on the radial distance from the epicenter (see Fig. 5 panel a).

We now investigate how the radial spread evolves over time (see Fig. 5 panel b). We note that in the time points before the fire, the spread of abnormal traffic spikes does not depend on the distance outward from the Notre-Dame Cathedral. In the time-points immediately following the fire, there is a rapid shift in the dependency of the volume of traffic based on radius from the Cathedral (see Fig. 5 panel b). We note that the more intense the decaying behavior of the functions in the form of Eq. (6), the more the distribution of data approximates a radial behavior. Thus, to further quantify the radial pattern of the data and how this radial pattern relaxes, we use linear regression to calculate a slope for each snapshot function shown in Fig. 5 panel b. We then plot how the slope of these best-fits lines change in time, with more negative slopes corresponding to more intense radial spread, which relaxes over time (see Fig. 5 panel c).

We also measure the KL divergence between the distribution of data in each snapshot of the form of Eq. (6) and the mean of the these snapshot distributions in the two hours following the fire (see Fig. 5 panel d). This describes how each snapshot behaves with respect to the average over the two hour interval, highlighting the striking difference between the behavior of the distributions before and following the fire, and emphasizing the persistence of the pattern in response to the fire into the night.

We then use the empirical abnormal traffic distribution data to construct a null model with optimal radial spread at each snapshot in time (see Methods). We measure the KL divergence at each time-point between the function in the form of Eq. (6) constructed from the data and the same functional form given by the optimally radial null model. This tells us how radial the reaction pattern in response to the catastrophe is with respect to its most radially possible distribution of the empirical measurements.

We observe that in the time points immediately preceding the fire, the distribution of the data is far from the distribution of the radial null model, showing a lack of dependence of abnormal traffic data on the epicenter of the fire. We then find a dramatic decrease in the divergence from the radial null model in the time points immediately following the fire, followed by a gradual rise in divergence as the intensity of the response relaxes. However, we note that the approximation to maximum radial spread remains prevalent until the end of the day (see Fig. 6).

This is an exemplar result, as one might have previously assumed that as it is possible to receive information of catastrophe at the same time, application traffic spikes would be distributed uniformly with respect to the epicenter. However, the spread of abnormal traffic volume in response to the fire at Notre-Dame moves outwards from the epicenter, like a real-world fire.

This result is in fact consistent with results from [22], who found a similar radial spread when looking at call traffic volume in response to catastrophic events such as earthquakes. The traffic intensity in those cases radiates outward in an radial way and the function in the form of Equation (6) aggregated over a two hour period hours approximates an exponential decay.

We note that in this study, our focus on Twitter is due to its large response and sustained activity, emphasizing radial patterns as we develop our analysis. We can also observe a similar radial spread in other applications. For example, Periscope and Whatsapp experience radial patterns (See Supplementary Fig. 16). Aligning with the time series analysis, the spreading radial pattern decays faster for Periscope. Similarly, the radial pattern is sustained until late in the evening for WhatsApp, with an overall above-baseline traffic lower than for the application Twitter.

#### Lyon, a further study of spatial spreading patterns

We now focus on a second catastrophic event, a bombing in Lyon, France, that occurred within the time period included the NetMob2023 dataset. We extend our spatial analysis of spiking traffic volume of the application Twitter in response to this event as a first test of the generalizability of the methodology we have proposed.

The Lyon bombing occurs at approximately 17:28 on Friday, May 24th of 2019 [28]. We plot abnormally high Twitter application traffic volume in Lyon over several time points in Fig. 7. Abnormally high traffic volume is measured with respect to baseline statistics calculated from all Friday’s prior to the bombing within the dataset. Unlike in the Twitter traffic in Paris during the Notre-Dame fire, it is not plainly evident by inspection a clear radial spatial spread outward from the epicenter of the catastrophe. Therefore, we apply our previous spatial analysis methods to more thoroughly describe the spatial propagation of information (see Fig. 8).

We find that in the two hours following the catastrophic event above-baseline traffic volume also experiences a dependence on the radial distance with respect to the epicenter of the catastrophe (see Fig. 8 panel a). This is consistent with what we found for the fire at Notre-Dame.

We look at this pattern at each time-point to further examine the reaction of application traffic to the event and assess the excitation and relaxation of information spread. Taking functions of the form of Eq. (6) for each time point, we can see the appearance of a radial pattern after the event, followed by a quick relaxation of this radial pattern in time (see Fig. 8 panel b). We again quantify this with slopes of linear regressions on each snapshot, showing the evolution of the intensity of radial spread in the time following a catastrophe and its relaxation in time (see Fig. 8 panel c).

Finally, we calculate the KL divergence between the data distribution in each snapshot according to Eq. (6) and the mean of these distributions in the two hours after the bombing (see Fig. 8 panel d). The KL divergence of the data to the 2 hour average is far noisier than the catastrophe of the fire of Notre-Dame. While there is consistency with the mean in the time-points immediately following the fire, the duration of this consistency is far shorter that of the catastrophe in Paris.

We posit that the differences we see between the duration of the radial spread in Paris and Lyon is due to the different duration of the two events. The cathedral fire occurs visibly over several hours, including the collapse of the historic spire and with continuous updates as firefighters battled the blaze. On the other hand, the Lyon bombing was a singular event that lasted only moments and was therefore not as conducive to live-streaming on social media as the hours-long fire at Notre-Dame.

We then repeat the radial spread null model and KL divergence analysis to asses how closely the response pattern to the catastrophe represents a spatially radial spread (see Fig. 9). We again find that following the bombing, the empirical pattern closely approximates the null model of radial distribution, consistent with what we observed in Paris. However, the different duration over which the two events unfold drastically affect the time of relaxation to the generally noisy baseline of the traffic volume after the two events.

Finally, we note that on the day of the catastrophe, there was a large planned event in Lyon, an Ed Sheeran concert, which was advertised to begin at 18:00 [29]. In Fig. 10, we demonstrate the extension of the methods of abnormal application traffic and pattern detection to planned events, and the interaction between two events. We note that we slightly modify our radial shell method to consider only the tiles *between* the two events *within* the city, until reaching the other event. Our results suggest a slight interaction between the traffic of the two events, however, this is mitigated by the large distance between the concert venue and the site of the bombing.

## Discussion

In this work we analyze time series of spatially distributed mobile phone applications and investigate the deviation from baseline activity in such traffic triggered by the fire of the Notre-Dame cathedral that took place on Monday, 15th April 2019. We use the Netmob2023 Data Challenge Dataset to investigate patterns of use across applications, study how these patterns vary within the city of incidence as opposed to other cities, and examine how the abnormal traffic volume propagates in space and time.

We introduce a definition of *spike* (see Methods) of anomalous application traffic and detect the presence of such spikes for apps in the cities of Paris, Marseille, Lyon, Montpellier, Rennes and Strasbourg, on the day of and the day after the fire of Notre-Dame. We note that applications that spike in other cities are a subset of applications that spike in Paris.

**Figure 1:**
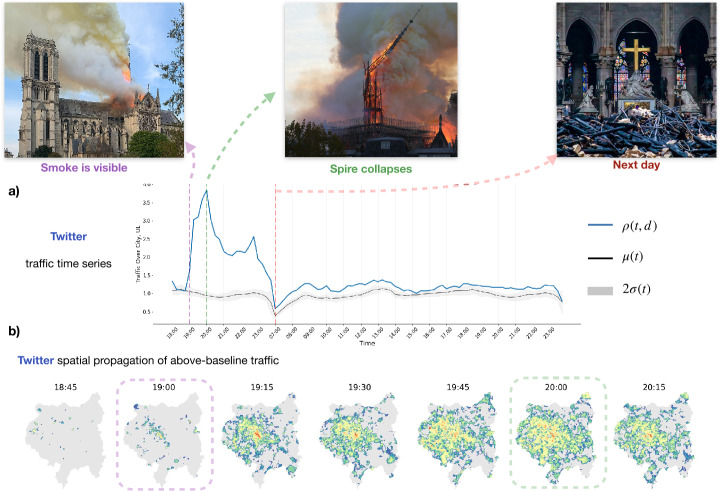
*Graphical introduction:* *a)* The time series evolution of the application Twitter from 18: 00 to end of day on the day of the fire and 7: 00 to end of day the day following the fire. The traffic on the day of the fire, *ρ*, is given in blue, and the mean (*μ*) and two standard deviations (2*σ*) of traffic in previous weeks is in grey. *b)* The spatial evolution of application traffic of Twitter is shown in time on the outline of the city of Paris, with intensity of app traffic from an established baseline displayed

The categories of apps whose time series of distance-from-baseline traffic have at least one spike include: social media, video streaming, and messaging platform. Interestingly, applications with the same function, such as social media, may exhibit differences in behavior. For example, Instagram follows a similar behavior patter across all cities, characterized by short (low duration) traffic spikes across all cities. However, Twitter, another social media network, is characterised by long and sustained spikes in traffic in time (see Fig. 1). This could be due to the tendency of users to rely on Twitter more than Instagram as a source of news and information on ongoing events [3].

**Figure 2:**
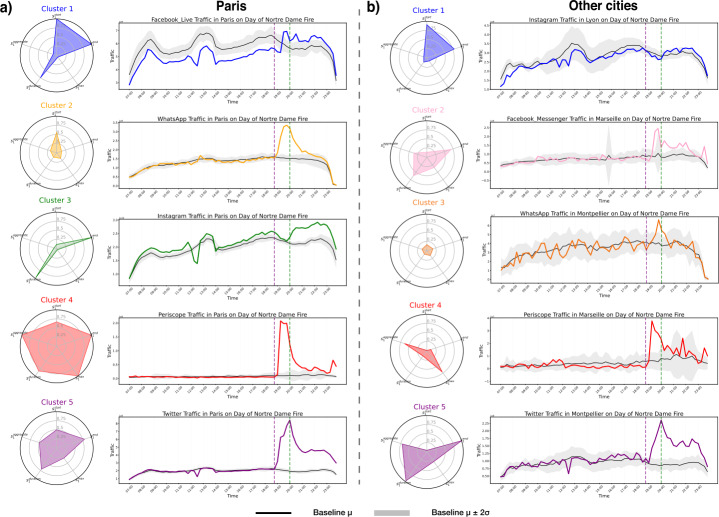
*Clusters and Representative Time Series on Day of Fire:* *a)* For the city of Paris, the radar plots of clusters of applications are given on the left, showing the value of different features assigned to each cluster. On the right of each radar plot is a time series for an application that is representative of the applications in that cluster. Note, the information in the radar plots for each cluster is normalized within the city of Paris. The applications in each cluster are given in Table 2. *b)* For all other cities being considered, the radar plots of clusters of applications are given on the left, with a representative time series of an of an application in the cluster shown on the right. Note, the information in the radar plots for each cluster is normalized for all cities excluding Paris. The applications in each cluster are given in Table 3. In each plot of the representative time series, the black solid line corresponds to the time series of the city-wise, average traffic volume $\mu (t)$ of the corresponding app, while the shaded area corresponds to the interval $\mu (t)\pm 2\sigma (t)$

**Figure 3:**
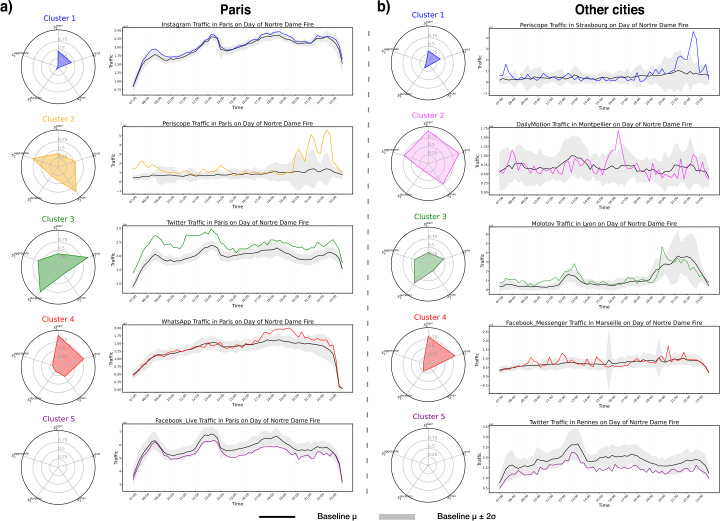
*Clusters and Representative Time Series on Day after Fire:* *a)* For the city of Paris, the radar plots of clusters of applications are given on the left, showing the value of different features assigned to each cluster. On the right of each radar plot is a time series for an application that is representative of the applications in that cluster. Note, the information in the radar plots for each cluster is normalized within the city of Paris. The applications in each cluster are given in Table 4. *b)* For all other cities being considered, the radar plots of clusters of applications are given on the left, with a representative time series of an of an application in the cluster shown on the right. Note, the information in the radar plots for each cluster is normalized for all cities excluding Paris. The applications in each cluster are given in Table 5. Note that apps in Cluster 5, both in *a* and *b* correspond to apps that spike on the day of fire, but do not spike on the day after the fire. In each plot of the representative time series, the black solid line corresponds to the time series of the city-wise, average traffic volume $\mu (t)$ of the corresponding app, while the shaded area corresponds to the interval $\mu (t)\pm 2\sigma (t)$

**Figure 4:**
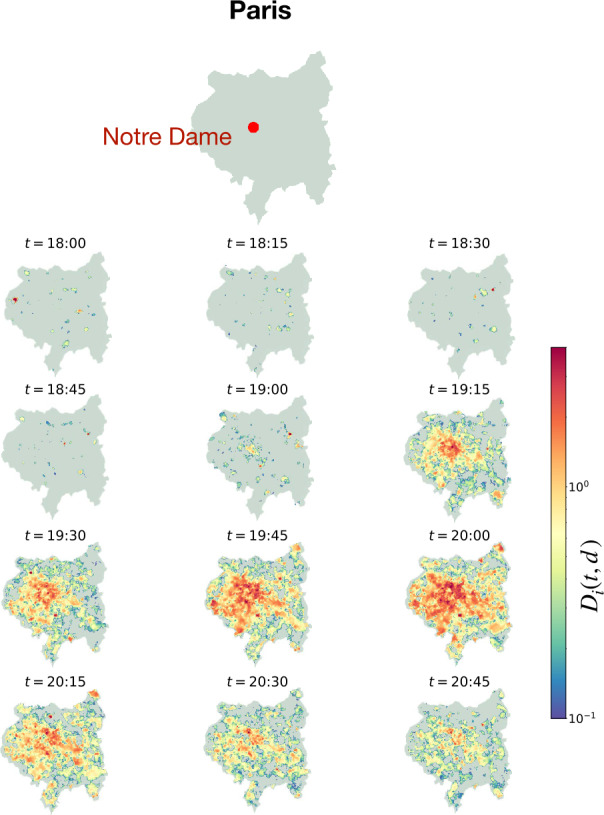
*Map of Paris with Abnormal Twitter Traffic:* The outline of the metropolitan area of Paris is shown at the top, with a red dot indicating the position of the Notre-Dame Cathedral. Below, each 15-minute timestamp shows the same city outline, now with each spatial tile as defined by the dataset displaying its corresponding tile-wise distance, $D_{i}(t)$, from baseline traffic. Only $D_{i}(t) > 0$ is considered. Recall that the first time-point considered to be related to the fire of Notre-Dame is 18:45

**Figure 5:**
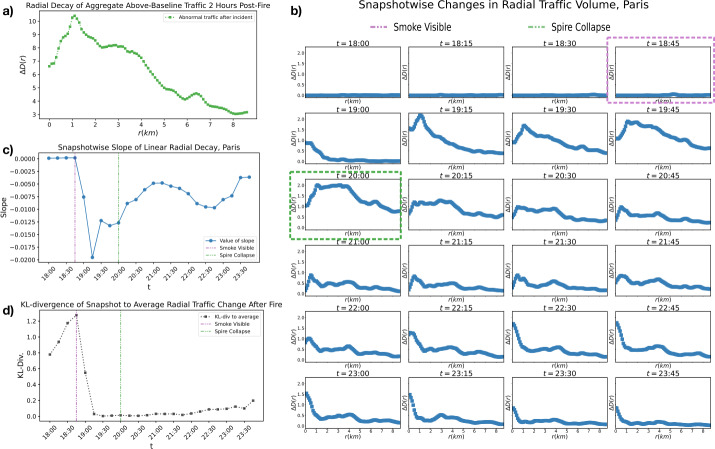
*Instantaneous and Aggregate Radial Patterns in Paris in Response to the Fire in Paris:* *a)* The change in abnormal traffic volume as a function of radius from the epicenter of the catastrophe (see Equation (6)) is shown over the course of two hours (between 18: 45 and 20: 45), showing the course grained, general behavior of spatial Twitter traffic response in the aftermath of the fire. The generally decreasing behavior of the function (particularly from 1 km) implies a radial spread of information. *b)* Changes in abnormal traffic volume with respect to distance from epicenter are shown at each 15-minutes time interval. From these snapshots, we can then track changes in the “radial-ity” of information spread. *c)* The slope of the best-fits line to each snapshot over time. This shows how the strength of radial spread changes over time. That is, a more radial distribution of information outward from the epicenter of the fire corresponds to a more negative slope, or a faster decay of the change in aggregate traffic from the epicenter. *d)* The Kullback-Leibler divergence of each snapshot in panel b to the average of snapshots from 18: 45 to 20: 45, showing how the strength of the radial spread of information varies with respect to its average change over the defined interval at each time-point. This emphasizes the change in traffic spread before and after the fire and the maintaining of the radial pattern throughout the rest of the night

**Figure 6:**
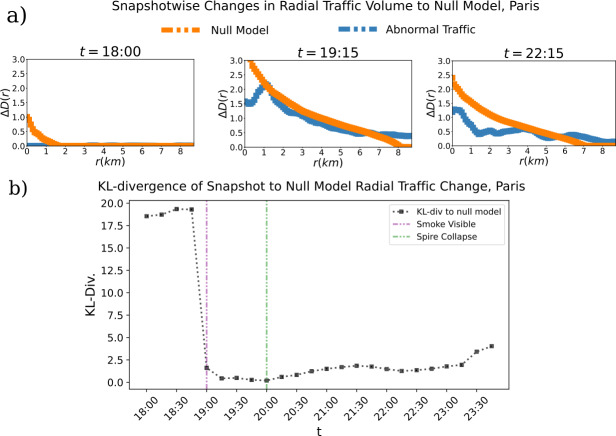
*Data vs. Null Model in Twitter Response to the Fire of Notre-Dame:* *a)* The change in abnormal traffic for the data and the null model at that respective time points is shown for 3 time points. Note that the first recorded time point after fire is 18: 45. *b)* The Kullback-Leibler divergence of each snapshot to the null model of most radial possible spread constructed at that time point is shown

**Figure 7:**
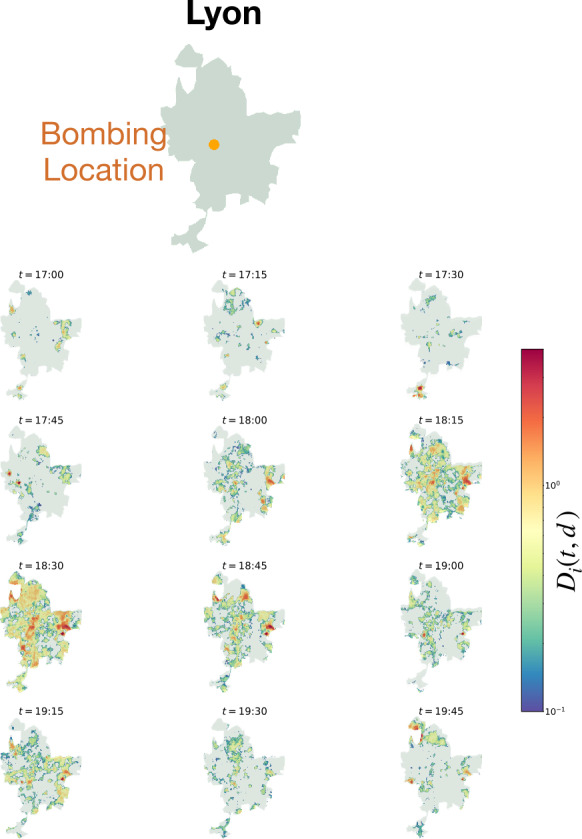
*Map of Lyon with Abnormal Twitter Traffic:* The outline of the metropolitan area of Lyon is shown at the top, with a red dot indicating the site of the bombing. Below, each 15-minute timestamp shows the same city outline, now with each spatial tile as defined by the dataset displaying its corresponding tile-wise distance, $D_{i}(t)$, from baseline traffic. Only $D_{i}(t) > 0$ is considered. Recall that the first time-point considered to be related to the bombing is 17:30

**Figure 8:**
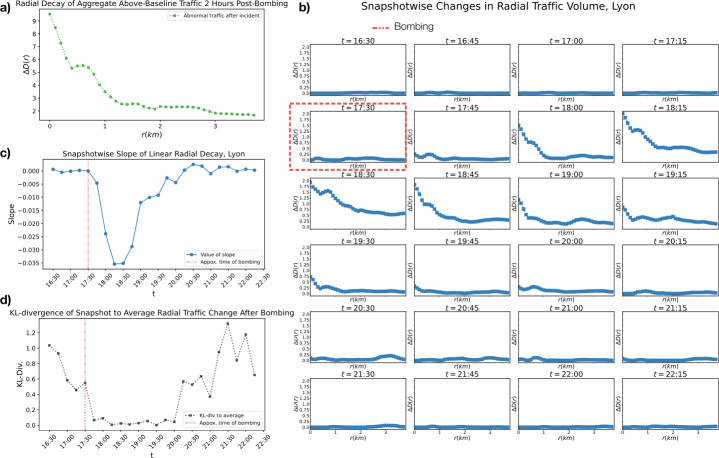
*Instantaneous and Aggregate Radial Patterns in Twitter Response to Bombing in Lyon:* *a)* The change in abnormal traffic volume as a function of radius from the epicenter of the catastrophe (see Equation (6)) is shown over the course of two hours (between 17: 30 and 19: 30), showing the course grained behavior of spatial Twitter traffic response in the aftermath of the bombing. The generally decreasing function implies a radial spread of information. *b)* Changes in abnormal traffic volume with respect to distance from epicenter are shown at each 15-minutes time interval. *c)* The slope of the best-fits line to each snapshot over time. This shows how the strength of radial spread changes over time. *d)* The Kullback-Leibler divergence of each snapshot in panel b to the average of snapshots from 17: 30 to 19: 30, showing how the strength of the radial spread of information varies with respect to its average change at each time-point

**Figure 9:**
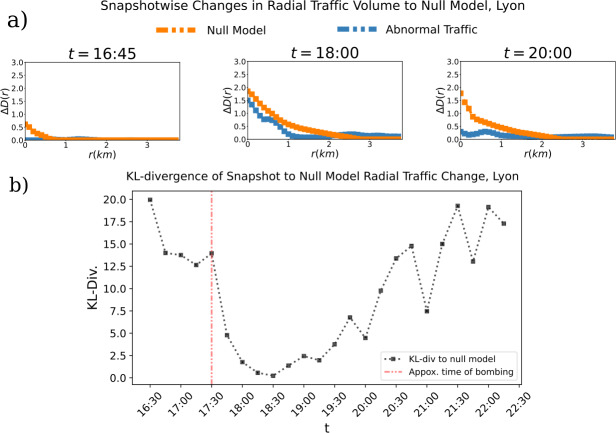
*Data vs. Null Model in Twitter Response to Bombing:* *a)* The change in abnormal traffic for the data and the null model at that respective time points is shown for 3 time points. Note that the first recorded time point after the bombing is 17: 30. *b)* The Kullback-Leibler divergence of each snapshot to the null model of most radial possible spread constructed at that time point is shown

**Figure 10:**
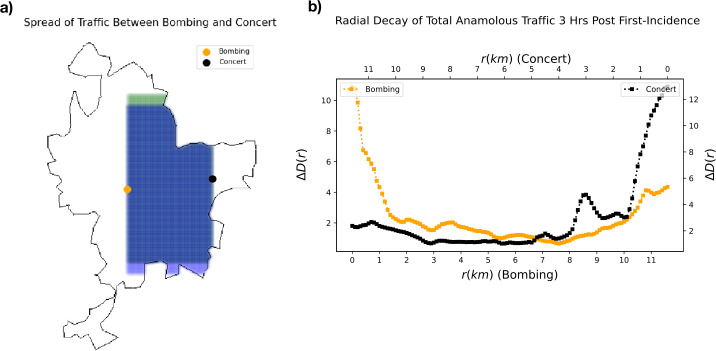
*Traffic overlap between bombing and concert in Lyon:* *a)* The outline of the metropolitan area of Lyon is shown, with a orange and black dots indicating the locations of the bombing and concert, respectively. The abnormal traffic between these two location *within* the city is considered, using squares of increasing radial size. *b)* The change in aggregate of abnormal traffic between the two epicenters of both the bombing and the concert are shown, from both epicenters towards the other epicenter

To further investigate these differences in the above-baseline traffic of apps of similar function, we investigate how applications cluster together based on the spiking patterns in time over the interval of interest. We separate the day of incidence and the day after to best capture the long term behavior of applications and how these may differ. We also perform the clustering separately for apps that spike in Paris and apps that spike in the other 5 cities to further investigate how patterns may differ in a city of incidence.

We find that between Paris and other cities on the day of interest, the applications experience generally similar trends in clustering, however, there is some diversity in the spiking patterns. For example, in application clustering can be heavily influenced by user-preference within a city. Further since broad application categories, such as social-media, cannot fully capture the diversity of application traffic response, it seems that we may need a more fine-grained look at function of applications, such as broadcasting and discussion based labeling. Distinct behaviors can be seen in Live Video streaming platforms, where short but intense spikes are seen. Also notable is the discussion based platform Twitter, which experiences a high and sustained spike. Most applications experience a fast relaxation from spiking, given that the next day many applications do not experience spikes in application traffic above baseline activity. This is especially true in cities that are not the city of incidence, showing that general interest in the event is more strongly maintained in the city of incidence.

The clustering of the time series of distance-from-baseline of app traffic in the other cities highlights how the effect of the event on an app is different across cities. Nonetheless, we note how live-streaming and, in general, video streaming apps such as Periscope, DailyMotion and Molotov, while with a delay with respect to Periscope in Paris, display a similar early spiking trend. We speculate that the delay in time of the anomalous traffic activity could be correlated with the physical distance from Paris, as well as to the time it takes for the video and pictures of the flaming cathedral to be noticed on these platforms in other cities.

The longest lasting, most sustained spikes tend to occur in Twitter in all of the cities. Such a result suggests that Twitter might be a suitable candidate to investigate the spatio-temporal spreading of the event-triggered anomalous traffic in the city of Paris. We thus investigate the existence of a relationship between how information propagates in the virtual information sphere, and how such propagation unfolds in physical space. We find that the anomalous, above-baseline activity emanates outward of in the immediate proximity of the cathedral of Notre-Dame. We further compare the data to a null model of optimal radial spread, demonstrating the shifts to radial data patterns in response to the fire and the relaxation from radial patterns over time. This result is surprising in that the intensity of the spread appears to decay radially with the distance from the epicenter of the catastrophe, which is not immediately evident given the means of communication being investigated, that, in principle, do not rely on physical distance. This result is consistent with other studies showing that call traffic volume radiates outward from the epicenter of catastrophe [22]. We further explore the spatial spread and its relaxation through a further analysis of a bombing in the city of Lyon. We find consistent results, however, the relaxation of abnormal traffic patterns in Lyon is much quicker than the fire of Notre-Dame in Paris, likely due to the much shorter duration of the event.

There are a number of possible reasons for the radial spread effect we observe here. For example, Twitter posts are often geo-tagged, and may suggest posts to users based on their location. Furthermore, the structure of the social network itself may have a geo-spatial relationship, as social networks have been shown to be influenced by both geographic proximity and residential segregation [30]. Increased data granularity and information about the social network structure would be necessary for a deeper understanding of this radial spread effect. Finally, we show planned events may also exhibit radial spreading patterns and suggest that events may interact with each other.

In conclusion, we provide a description of how catastrophic events, particularly the fire of the Notre-Dame cathedral perturb the temporal and spatial patterns of app usage traffic. An interesting development of the current research would be to characterise the spreading of the above-baseline activity at a coarser spatial scale, thus considering entire cities as spiking tiles. Then, a definition of spiking delay with respect to Paris could be introduced, in order to investigate whether any spatio-temporal patterns could be found. We speculate that such result could be achieved with a more granular time resolution, as, potentially, other means of communication, such as news broadcasting, could enforce a synchronisation of the spiking activity in the other French cities.

## Supplementary Information

Below is the link to the electronic supplementary material.

(PDF 3.2 MB)

## Data Availability

Access to the NetMob23 dataset was granted under a licensing agreement as part of the NetMob 2023 Data Challenge. Further information is available at https://netmob2023challenge.networks.imdea.org/. A full description of the dataset used in this work can be found in https://arxiv.org/abs/2305.06933.

## Abbreviations

KL divergence or KL-div.: Kullback-Leibler Divergence
Pa: Paris
Mrs: Marseille
Ly: Lyon
Mtp: Montpellier
Rn: Rennes
Sg: Strasbourg
