# Supplementary material for: Detection of anomalous spatio-temporal patterns of app traffic in response to catastrophic events
Source: EPJ Data Sci. 2025 May 6;14(1):35. doi: 10.1140/epjds/s13688-025-00546-w (PMC12055615; doi:10.1140/epjds/s13688-025-00546-w)
Supplement: Supplementary file 1 — (PDF 3.2 MB) [file 13688_2025_546_MOESM1_ESM.pdf]

5 Supplementary Materials

5.1 Applications under consideration

Our analysis considers the applications represented in the NetMob2023 Data Challenge dataset. The description of the 68 available applications is given in [23]. We first sort for relevance, with our criteria for included apps consisting social media, messaging, transportation and location, video-sharing and streaming, and cloud storage apps.

We recover the following list of 24 applications that will be assessed for traffic spikes:

- Apple App Store
- Apple Video
- Apple iCloud
- Apple iMessage
- DailyMotion
- Facebook
- Facebook Live
- Facebook Messenger
- Google Drive
- Google Maps
- Google Play Store
- Instagram
- Molotov
- Orange TV
- Periscope
- Snapchat
- Telegram
- Twitch
- Twitter
- Uber
- Waze
- Web Transportation
- WhatsApp
- YouTube

Of the applications considered, 11 of 24 applications experience spike. The full table of all spiking app is found in Table 1 in the main text. None of the transportation or location applications considered experience abnormally high traffic spikes. Other applications that do not experience traffic spikes include the messaging app Telegram and the video streaming app Youtube.

5.2 Feature Values

We include features that describe the commencement time, duration, and intensity of the application traffic spike. This includes the start and end time of the each anomalous traffic spike, as well as the magnitude of deviation from baseline traffic (see Methods for more details).

5.2.1 Features, day of fire

A full description of the non-normalized values for the features of spiking applications on the day of the fire of Notre Dame is given in Tables 6 and 7. Table 6 shows the non-normalized values for features of applications in the city of Paris and Table 7 shows the values for the features of spiking applications in other cities. The distribution of the values of each feature across all applications is other cities on the day of the fire is shown in Figure 11.

App	s_1^{start}	s_1^{end}	s_1^{max}	$s_1^{duration}[hrs.mins]$	$s_1^{aggregate}$
Apple Video	19.15	21.15	2.10	2.00	8.42
Apple iCloud	18.45	23.45	0.40	5.00	3.54
DailyMotion	19.30	23.45	1.02	4.15	8.05
Facebook	19.00	23.45	0.32	4.45	3.52
Facebook Live	19.30	23.45	0.11	4.15	0.51
Facebook Messenger	19.15	22.45	3.54	3.30	15.29
Instagram	18.45	23.45	0.24	5.00	2.71
Molotov	19.15	21.45	2.28	2.30	9.43
Periscope	19.15	23.45	9.60	4.30	41.34
Twitter	19.00	23.45	2.93	4.45	25.95
WhatsApp	19.00	21.45	1.00	2.45	4.68

Table 6 Non-normalized features values for applications spiking in Paris on the day of fire.

City	App	s_1^{start}	s_1^{end}	s_1^{max}	$s_1^{duration}[hrs.mins]$	$s_1^{aggregate}$
Marseille	Facebook Messenger	19.30	22.30	0.98	3.00	3.28
	Periscope	19.15	20.45	1.95	1.30	4.83
	Twitter	19.15	23.45	0.99	4.30	7.76
Strasbourg	Apple Video	19.45	20.30	0.36	0.45	0.53
	DailyMotion	19.45	21.00	0.47	1.15	0.93
	Facebook	20.00	21.00	0.09	1.00	0.17
	Facebook Messenger	19.15	22.00	2.04	2.45	6.38
	Molotov	19.45	20.30	0.63	0.45	1.26
	Periscope	19.15	20.15	1.97	1.00	4.74
	Twitter	19.15	23.45	0.92	4.30	4.35
Lyon	Apple Video	19.45	20.45	1.04	1.00	2.49
	Facebook Messenger	19.30	21.45	1.77	2.15	5.33
	Instagram	22.00	23.45	0.03	1.45	-0.06
	Molotov	19.30	21.00	1.01	1.30	2.75
	Periscope	19.15	21.00	3.67	1.45	9.80
	Twitter	19.15	23.45	1.52	4.30	10.89
Montpellier	Apple Video	19.45	21.00	0.70	1.15	2.27
	DailyMotion	19.45	23.45	1.18	4.00	6.01
	Facebook	19.45	23.45	0.13	4.00	0.95
	Facebook Messenger	19.30	22.30	1.81	3.00	5.14
	Instagram	20.15	23.45	0.23	3.30	1.58
	Molotov	20.00	21.00	0.40	1.00	0.66
	Periscope	19.15	20.30	2.91	1.15	8.22
	Twitter	19.15	23.45	1.32	4.30	9.29
	WhatsApp	19.45	20.30	0.26	0.45	0.48
Rennes	Facebook Messenger	19.30	22.30	1.88	3.00	5.17
	Periscope	19.15	20.15	1.23	1.00	2.12
	Twitter	19.15	21.15	0.61	2.00	2.16

Table 7 Non-normalized feature values for applications spiking in other cities on the day of fire.

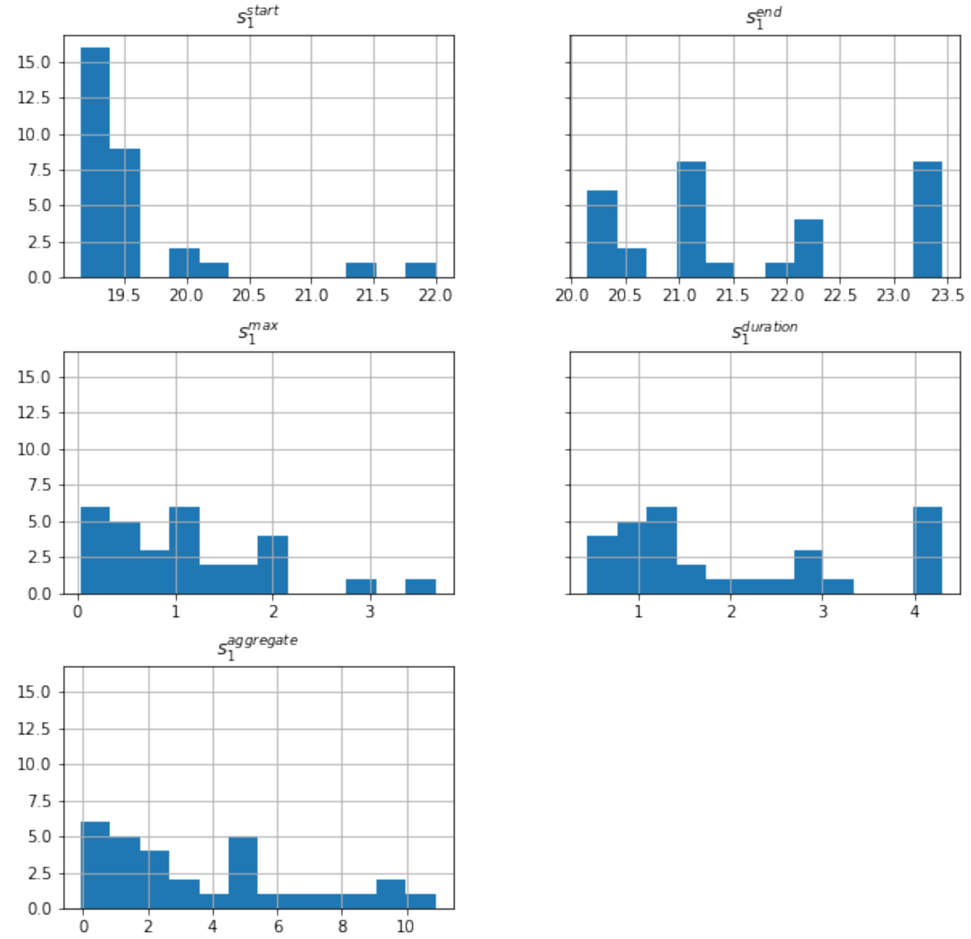

Fig. 11 Distributions of feature values, day of: The distribution of the values of each feature for all other cities on the day of the fire is shown.

5.2.2 Features, day after fire

A full description of the non-normalized values for the features for the day of the after fire of Notre Dame is given in Tables 8 and 9. Table 8 shows the non-normalized values for features of applications in the city of Paris and Table 9 shows the values for the features of spiking applications in other cities. The distribution of the values of each feature across all applications is other cities on the day after fire is shown in Figure 13.

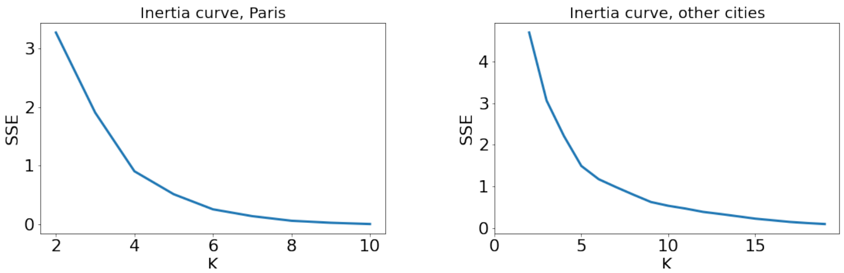

Fig. 12 K-means clustering, day of: Inertia of the K-means clustering of the app-spiking features computed on the day of the fire as a function of the number of clusters K for Paris (left) and other cities (right).

App	s_2^{start}	s_2^{end}	s_2^{max}	$s_2^{duration}[hrs.mins]$	$s_2^{aggregate}$
Apple Video	7.00	20.45	0.57	13.45	10.60
Apple iCloud	7.15	8.00	0.07	0.45	0.10
DailyMotion	15.45	17.15	1.01	1.30	4.24
Facebook	9.00	11.45	0.03	2.45	0.18
Facebook Live	0.00	0.00	0.00	0.00	0.00
Facebook Messenger	0.00	0.00	0.00	0.00	0.00
Instagram	8.15	9.45	0.04	1.30	0.11
Molotov	7.00	16.15	1.42	9.15	14.67
Periscope	7.00	9.00	1.72	2.00	8.89
Twitter	7.00	23.45	0.33	16.45	7.88
WhatsApp	17.30	20.45	0.15	3.15	1.04

Table 8 Non-normalized features values for applications spiking in Paris on the day *after* the fire.

5.3 Joint Two-Day clusters

We present the joint two-day clustering of application behavior in response to the fire of Notre-Dame. In contrast to the analysis presented in the main text, which classifies short and long term behavior of applications as separate behavioral patterns, this section considers application traffic response over an extended period of time. We define two spikes in abnormal traffic volume for an consistent with our previous analysis (see Methods for further details), recovering a 10 dimensional features vector for each spiking application. This 10 dimensional vector is composed of the union of the 5 dimensional features vectors for an application across the day of and day after the fire. We perform a K-means clustering on these features vectors after normalization, computing clustering for applications in Paris and in other cities separately, recovering 6 clusters for Paris and 5 clusters for other cities (see Figure 15).

City	App	s_2^{start}	s_2^{end}	s_2^{max}	$s_2^{duration}[hrs.mins]$	$s_2^{aggregate}$
Marseille	Facebook Messenger	9.30	10.15	0.24	0.45	0.45
	Periscope	22.15	23.00	0.45	0.45	1.17
	Twitter	0.00	0.00	0.00	0.00	0.00
Strasbourg	Apple Video	0.00	0.15	0.12	0.15	0.12
	DailyMotion	16.00	17.00	0.44	1.00	1.56
	Facebook	0.00	0.00	0.00	0.00	0.00
	Facebook Messenger	0.00	0.00	0.00	0.00	0.00
	Molotov	0.00	0.30	0.25	0.30	0.47
	Periscope	21.45	23.00	1.71	1.15	3.61
	Twitter	0.00	0.00	0.00	0.00	0.00
Lyon	Apple Video	7.30	8.30	0.16	1.00	0.30
	Facebook Messenger	0.00	0.00	0.00	0.00	0.00
	Instagram	0.00	0.00	0.00	0.00	0.00
	Molotov	8.00	12.15	0.92	4.15	3.99
	Periscope	7.15	8.45	0.36	1.30	0.57
	Twitter	0.00	0.00	0.00	0.00	0.00
Montpellier	Apple Video	7.45	8.30	0.26	0.45	0.35
	DailyMotion	16.15	17.00	0.50	0.45	0.63
	Facebook	21.45	23.45	0.10	2.00	0.19
	Facebook Messenger	0.00	0.00	0.00	0.00	0.00
	Instagram	0.00	0.00	0.00	0.00	0.00
	Molotov	7.00	8.45	1.27	1.45	4.05
	Periscope	14.15	15.30	0.40	1.15	0.54
	Twitter	8.00	13.30	0.24	5.30	1.17
	WhatsApp	0.00	0.00	0.00	0.00	0.00
Rennes	Facebook Messenger	0.00	0.00	0.00	0.00	0.00
	Periscope	21.30	23.00	3.34	1.30	7.15
	Twitter	0.00	0.00	0.00	0.00	0.00

Table 9 Non-normalized feature values for applications spiking in other cities on the day *after* the fire.

Cluster 1	Cluster 2	Cluster 3	Cluster 4	Cluster 5	Cluster 6
Apple iCloud	Apple Video	Periscope	Twitter	Facebook Live	DailyMotion
Facebook	Molotov			Facebook Messenger	WhatsApp
Instagram					

Table 10 Joint two-day clusters for Paris

Cluster 1	Cluster 2	Cluster 3	Cluster 4	Cluster 5
Periscope Mrs	Facebook Messenger Sg	Apple Video Sg	DailyMotion Sg	DailyMotion Mtp
Periscope Sg	Facebook Messenger Ly	Apple Video Ly	Molotov Ly	Facebook Mtp
Periscope Ly	Facebook Messenger Mtp	Apple Video Mtp	Molotov Mtp	Facebook Messenger Mrs
Periscope Mtp	Facebook Messenger Rn	Facebook Sg	Periscope Rn	Twitter Mtp
	Instagram Rn	Instagram Ly		
	Twitter Mrs	Molotov Sg		
	Twitter Sg	Twitter Rn		
	Twitter Ly	Whatsapp Mtp		

Table 11 Joint two-day clusters for other cities, Marseille (Mrs), Lyon (Ly), Montpellier (Mtp), Rennes (Rn) and Strasbourg (Sg)

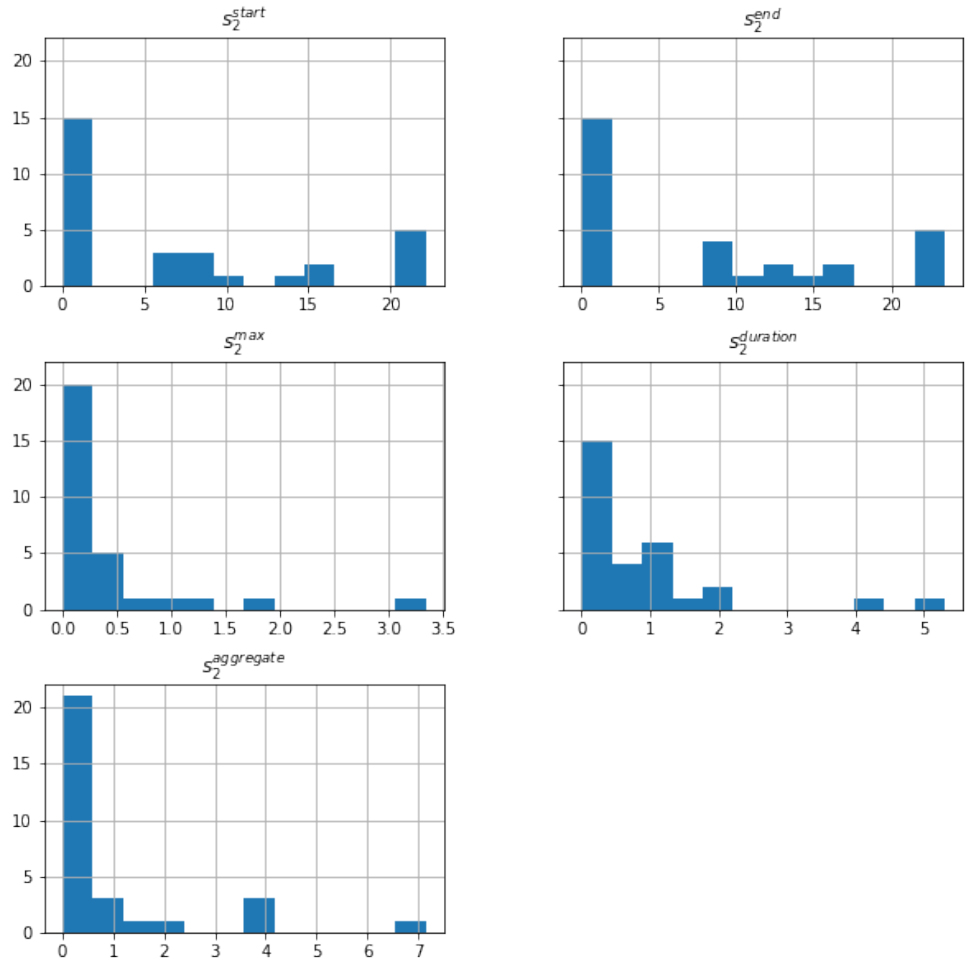

Fig. 13 Distributions of feature values, day after: The distribution of the values of each feature for all other cities on the day *after* the fire is shown.

In the joint two-day clusters, the short and long term behavior of applications are considered a single pattern. From the joint clusters, we find similar trends to those seen in the main text. For example, for Paris, Twitter and Periscope both form an outlier cluster with a single defined behavior, as is done on the day of the fire. Likewise, for other cities, we have a clustering consisting of only the app Periscope. Interestingly, for other cities the applications Twitter and Facebook Messenger, which have distinct behaviors when considered through the perspective of short and long term behaviors,

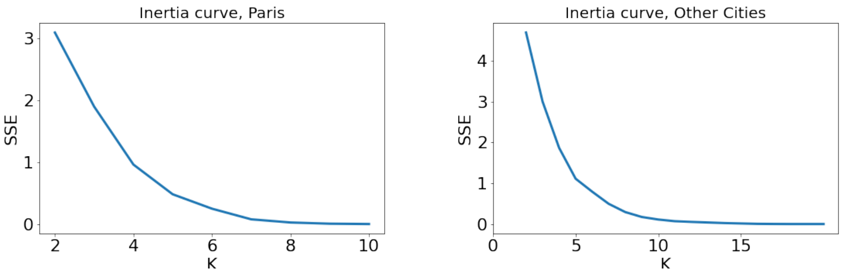

Fig. 14 K-means clustering, day after: Inertia of the K-means clustering of the app-spiking features computed on the day after the fire as a function of the number of clusters K for Paris (left) and other cities (right).

cluster together when considering the joint clustering. This is largely due to both of their abnormal traffic activity being largely confined to the day of the fire.

6 Spatial Spreading in Additional Applications

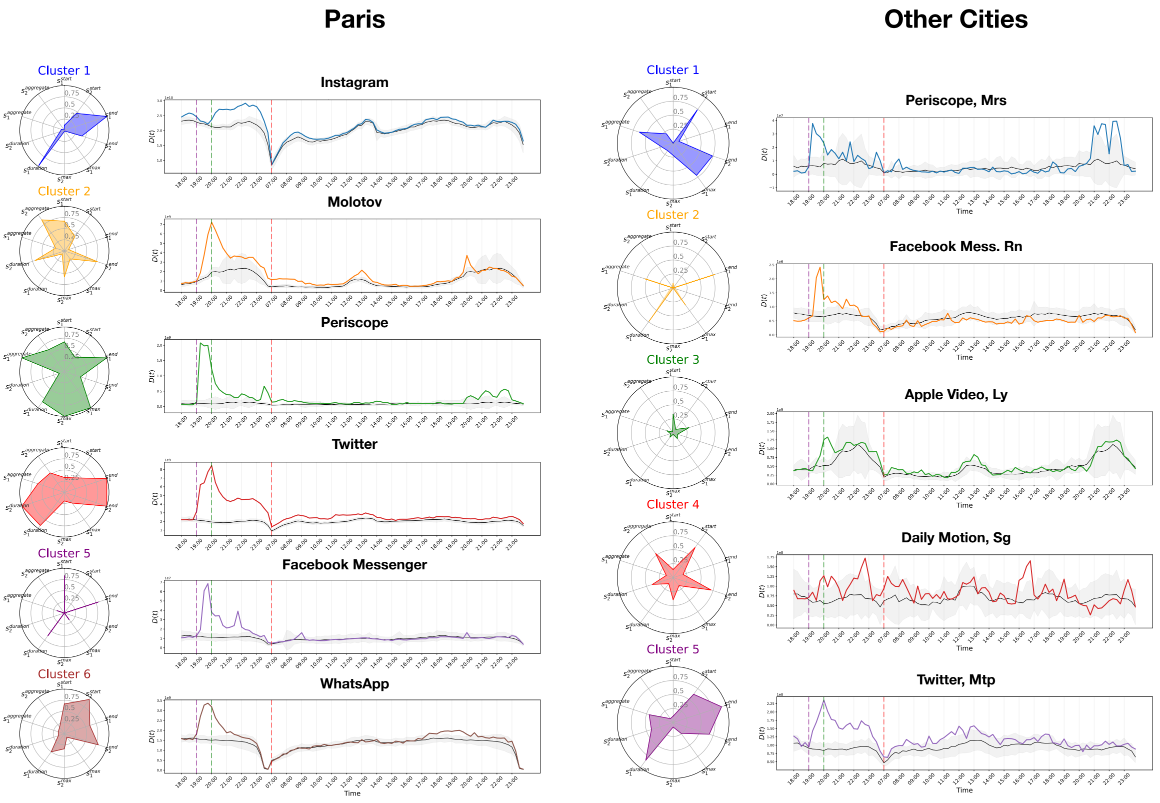

Fig. 15 Clusters and Representative Time Series Over Two Days: a) For the city of Paris, the radar plots of clusters of applications are given on the left, showing the value of different features assigned to each cluster. On the right of each radar plot is a time series for an application that is representative of the applications in that cluster. Note, the information in the radar plots for each cluster is normalized within the city of Paris. The applications in each cluster are given in Table 10. **b)** For all other cities being considered, the radar plots of clusters of applications are given on the left, with a representative time series of an of an application in the cluster shown on the right. Note, the information in the radar plots for each cluster is normalized for all cities excluding Paris. The applications in each cluster are given in Table 11.

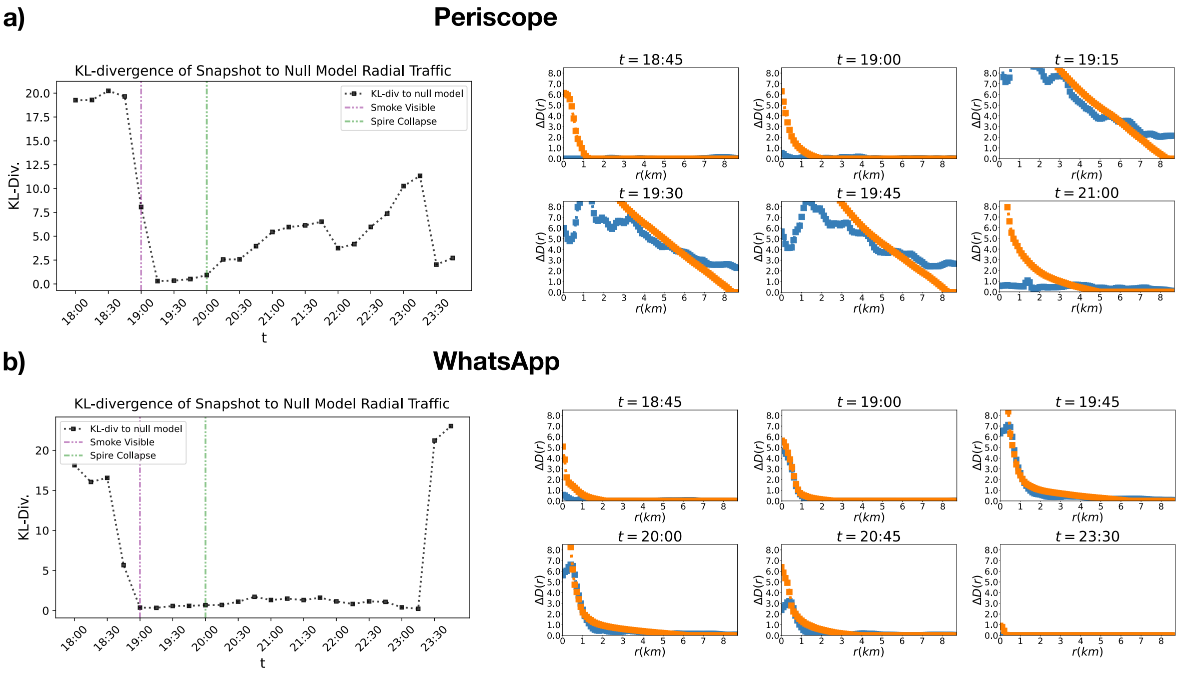

Fig. 16 Radial spreading patterns on other apps: a) The Kullback-Leibler divergence of each snapshot to the null model of most radial possible spread constructed as a function of time (left), for the app Periscope; The change in abnormal traffic for the data and the null model at that respective time points is shown for 6 time points. **b)** The Kullback-Leibler divergence of each snapshot to the null model of most radial possible spread constructed as a function of time (left), for the app WhatsApp; The change in abnormal traffic for the data and the null model at that respective time points is shown for 6 time points.
